# HIV prevalence and associated factors among women in urban Ethiopia: findings from the Ethiopian Population-based HIV Impact Assessment

**DOI:** 10.1016/j.ijregi.2026.100918

**Published:** 2026-05-22

**Authors:** Kidist Zealiyas, Yimam Getaneh, Terefe Gelibo, G/Medhin G/Michael, Sileshi Lulseged, Hailegnaw Eshete, Abebe Habteselassie, Getachew Tollera, Gemechu Tadesse, Minilik Demissie Amogne

**Affiliations:** 1 Ethiopian Public Health Institute, Addis Ababa, Ethiopia; 2 ICAP in Ethiopia, Mailman School of Public Health, Columbia University, Addis Ababa, Ethiopia

**Keywords:** EPHIA, Ethiopia, HIV Prevalence, Women

## Abstract

**Objectives:**

Women remain disproportionately affected by HIV in Ethiopia and worldwide due to biological, behavioral, and socioeconomic factors. This study describes HIV prevalence and associated determinants among women in urban Ethiopia.

**Methods:**

Data were drawn from the Ethiopian Population-based HIV Impact Assessment (EPHIA), a cross-sectional household survey conducted between October 2017 and April 2018. A two-stage cluster sampling approach was used to select households. Of 12,158 eligible women aged 15-64 years, 11,599 were included in the analysis. HIV testing followed the national algorithm. Associations were assessed using adjusted odds ratios (AORs) with 95% confidence intervals (CIs).

**Results:**

The overall HIV prevalence among urban women was 4.1%. The highest prevalence was observed in Gambella (8.0%), Harari (7.6%), and Dire Dawa (5.7%). Women aged 35-44 years (9.1%), widowed (15.1%), with no education (6.2%), or reporting multiple sexual partners (11.2%) had elevated prevalence. Adjusted analyses showed significantly higher odds of HIV among women aged 35-44 years (AOR 7.2; 95% CI: 3.9-13.5), those with no education (AOR 4.7; 95% CI: 2.2-10.2), widows (AOR 3.4; 95% CI: 1.6-7.0), and those who underwent genital cutting (AOR 3.4; 95% CI: 2.0-5.8).

**Conclusion:**

HIV remains a major public health challenge among urban Ethiopian women. Targeted interventions should prioritize older women, widows, and those with limited education to reduce disparities and advance national HIV control efforts.

## Highlights

• HIV prevalence among urban Ethiopian women is 4.1%.
• Highest rates found in Gambella, Harari, and Dire Dawa regions.
• Older age (35-44 years), widowhood, and no education increase HIV risk.
• Genital cutting significantly is associated with higher HIV prevalence.
• Findings support targeted interventions for vulnerable women.

## Background

HIV remains a major global public health challenge. In 2024, 40.8 million people were living with HIV, including 39.4 million adults and 1.4 million children aged under 15 years [1]. Women represent 53% of adults (15-49 years) living with HIV worldwide, and HIV is a leading cause of death among women of reproductive age (15-49 years) [1,2]. In sub-Saharan Africa, an estimated 26 million people lived with HIV in 2024, representing two-thirds of the global total and disproportionately affecting women, who comprised more than half of cases. Notably, young women aged 15-24 years accounted for 19% of new infections that year [[3], [4], [5]]. According to the 2025 Ethiopian Demographic Health Survey, the overall prevalence of HIV was 0.8%, 0.7% among men and 0.9% among women aged 15-49 years, concentrated in urban areas, and varied by administrative region [6].

HIV infections in Ethiopia were first documented in a young man and woman in 1984 in Addis Ababa when serum samples of 167 hospitalized patients with Bell’s palsy were tested for anti-HIV antibodies [7].

Late 1980s, a high prevalence of HIV estimated at 17% was detected among commercial sex workers residing in urban areas [8]. Starting in 2003, the US President’s Emergency Plan for AIDS Relief became the second largest donor for HIVAIDS in Ethiopia, leading to the development of national prevention, care, and treatment guidelines; establishment of program structures and systems; and enhancement of human capacity through training and site-level support [9].

Women are disproportionally affected by HIV due to biological, social, behavioral, cultural, and economic factors [10]. Biological factors include molecular, genetic, and immunological variations that influence an individual’s susceptibility to the virus [11]. Lower education, unemployment, and economic and sex disparity among women are associated with higher rates of HIV infection [12]. Risk factors associated with HIV among women include early sexual debut, multiple sexual partners, no condom use, sexual and sex-based violence, and intergenerational sex. Socio-demographic factors such as age, marital status, level of education, employment, and place of residence have also been associated with the risk of HIV among young women [4,13]. This analysis aims to describe HIV prevalence and factors associated with HIV among women in urban Ethiopia.

## Methods

### Study design and population

The Ethiopia Population-Based HIV Impact Assessment (EPHIA) was a nationally representative, cross-sectional, household-based survey conducted in urban Ethiopia (Tigray; Afar; Amhara; Oromia; Somali; Benishangul Gumuz; Southern Nations, Nationalities, and Peoples’ Region (SNNPR); Gambella; Harari; Addis Ababa; and Dire Dawa) from October 2017 to April 2018 [14]. The study population for this analysis included women aged 15-64 years.

### Sample size determination

This study is a secondary analysis of data obtained from the EPHIA, a cross-sectional household survey conducted between 2017 and 2018.The sample for this analysis was derived from the parent EPHIA survey, which used a two-stage cluster sampling approach to ensure a representative sample of the urban population. From the total of 12,158 eligible women identified in the survey, 11,599 were included in this study after excluding participants with missing critical data points, such as HIV testing status or key demographic information [15]. The eligibility criteria for this study included all women aged 15-64 years who were residents of the selected households and provided informed consent for participation in the EPHIA survey.

### Study variables

The dependent variable for this analysis is HIV status among women aged 15-64 in urban Ethiopia. The independent variables were demographic characteristics (administrative region, marital status, educational level, age, employment status, and wealth quintile) and individual risk factors for HIV infection among women (condom use, first sex before 15 years of age, and number of sexual partners).

### Laboratory methods

Home-based HIV testing and counseling (HBTC) with immediate return of results were conducted in each household using the Ethiopian National HIV testing algorithm. The survey used a sequential rapid-testing algorithm in the field: the Wantai HIV 1/2 (Beijing Wantai Biological Pharmacy Enterprise Co., Ltd, Beijing, China) as a screening test, the Uni-Gold HIV 1/2 (Trinity Biotech, plc., Wicklow, Ireland) as a confirmatory test, and the Vikia HIV 1/2 (bioMérieux, SA, F-69280 Marcy l’Etoile, France) as a tie-breaker test. Individuals with a nonreactive result on the screening test were reported as HIV-negative and those with a reactive screening test underwent confirmatory testing. All specimens that tested HIV-positive during HBTC and those who had confirmed positive rapid test results during quality assurance underwent confirmatory testing using the Genius HIV 1/2 Supplemental Assay (Bio-Rad, Hercules, CA, USA). A positive Genius result defined HIV-positive status for the survey [16], HIV DNA (Deoxyribonucleic Acid) polymerase chain reaction (PCR) was performed at the Central Laboratory for infant virological testing and to confirm the status of respondents who self-reported an HIV-positive status but tested negative in HBTC. HIV incidence testing, antiretroviral (ARV) drug resistance testing, HIV viral load (VL) testing, and long-term storage of samples at −80°C also occurred at the Central Laboratory. Test results for HIV VL and HIV DNA PCR for infant virological testing were returned to the participants’ preferred health facility.

### Data collection procedures

The questionnaire was uploaded on tablets and administered to all eligible and consenting participants aged 15 years and older during face-to-face interview. Demographic, behavioral, and clinical data, including the home-based HIV testing data, were collected electronically in the field; details are provided in the full EPHIA report [15].

### Data analysis

We analyzed data on 11,599 women aged 15-64 years to determine the risk factors for HIV in urban women in Ethiopia. We examined various socio-demographic and behavioral factors, including age, marital status, education level, employment status, age at first sexual encounter, number of sexual partners, and condom use, all of which have been associated with HIV prevalence in women [4,17,18].

The analyses were conducted using Stata version 16 [19]. The primary outcome variable for the analysis was HIV status among women. Weighted proportions and 95% confidence intervals (CIs) were calculated to estimate the prevalence; jackknife weighing was applied in this analysis. HIV prevalence estimates were compared for different demographic and behavioral characteristics. All categorical variables were summarized using frequencies and proportions. Variables with chi-square *P* ≤0.05 were considered statistically significant. All variables achieving statistical significance in the bivariable analysis were included in the multivariable regression model.

## Results

**Figure 1:**
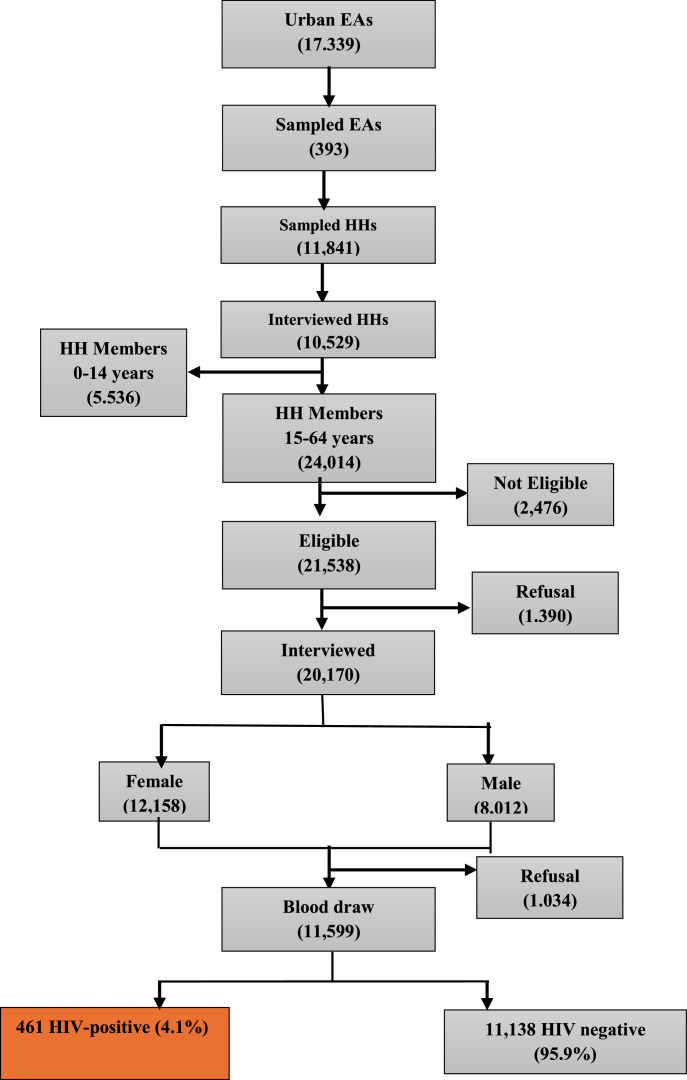
Participants selection flow diagram, Ethiopia Population-based HIV Impact Assessment (EPHIA) 2017-2018.

A total of 12,158 (96.3%) women consented and completed the adult interview; 11,599 (95.4%) had blood drawn. All blood samples were tested for HIV, of which 461 (4.1%) were HIV-positive (Figure 1).

### Demographic and behavioral characteristics of the study participants

A total of 85.4% of the women were from four administrative regions: Oromia, Amhara, SNNPR, and Addis Ababa (Table 1). A total of 50.8% were married, 55.4% had less than secondary education, 63.8%were not employed in the past 12 months, and 65.7%were in the age group 15-34 years. Ever having had sex was reported by 78.8% of women, of whom 8.4% reported first sex before age 15 years and 64.7% reported one sexual partner in the 12 months before the survey. In the preceding 12 months, only 4.4% of women used a condom at their last sex, 61.7% of women did not use a condom at their last sex, and 34% of women reported having no sex (Table 1). Furthermore, 78.3% of women did not use a condom at the last non-marital sexual intercourse (Table 1). Only 73.6% of women were ever previously tested for HIV, and among women positive for HIV, 93.4% were ever previously tested. Of the women positive for HIV, 83.3% knew their HIV status and 96.4% of women who knew their HIV status self-reported as currently on antiretroviral therapy (ART) or had detectable ARVs; 71.7% of women were virally suppressed and 69.3% (95% CI 64.4, 73.9) of women who knew their HIV status and were on ART were virally suppressed (Table 1). A total of 59% of women had undergone genital cutting, and 4% had experienced sexual violence (Table 1). Moreover, 99.1% of women had long-term infections, and 45.6% had their first HIV-positive test result 6-10 years ago (Table 1).

**Table 1:** Distribution of women aged 15-64 years by selected demographic and behavioral characteristics, Ethiopia Population-based HIV Impact Assessment (EPHIA) 2017-2018.

Characteristics	n	% (95% CI]*
**Region**		
Tigray	935	8.3[7.9-8.7]
Afar	497	1.3[1.2-1.4]
Amhara	1946	18.3[17.7-18.9]
Oromia	2837	33.5[32.7-34.3]
Somali	590	1.3[1.2-1.4]
Benishangul Gumuz	468	1.2[1.2-1.3]
SNNPR	1506	14.2[13.6-14.7]
Gambella	443	0.5[0.5-0.6]
Harari	448	0.7[0.6-0.7]
Addis Ababa	1948	19.4[18.8-20.0]
Dire Dawa	540	1.4[1.3-1.5]
Total (N)	12,158	100
**Sex of head of household**		
Male headed HH	4296	37[36.0-38.0]
Female-Headed Households	7862	63[62.0-64.0]
Total (N)	12,158	100
**Age group of adults**		
15-24 years	5004	34.9[33.9-35.8]
25-34 years	3600	30.8[29.8-31.8]
35-44 years	1930	18.5[17.6-19.4]
45-54 years	964	9.9[9.3-10.6]
55-64 years	660	6[5.5-6.5]
Total (N)	12,158	100
**Marital status**		
Never married	3977	29.3[28.4-30.2]
Married or living together	5890	50.8[49.7-51.8]
Divorced or separated	1473	12.9[12.2-13.6]
Widowed	737	7[6.5-7.6]
Total (N)	12,077	100
**Education level**		
No education	2068	18.1[17.3-19.0]
Primary	4464	37.3[36.3-38.4]
Secondary	3247	26.2[25.3-27.1]
More than secondary	2333	18.4[17.6-19.2]
Total (N)	12,112	100
**Wealth quintile**		
Lowest	2057	16.1[15.3-16.9]
Second	2059	16.4[15.6-17.1]
Middle	2358	19[18.2-19.8]
Fourth	2653	22.1[21.2-23.0]
Highest	3030	26.4[25.6-27.3]
Total (N)	12,157	100
**Employment in the past 12 months**		
Did not work in the last 12 months	7984	63.8[62.8-64.8]
Employed in the last 12 months	4155	36.2[35.2-37.2]
Total (N)	12,139	100
**Ever had sex**		
Never had sex	2935	21.2[20.4-22.0]
Have had sex	9118	78.8[78.0-79.6]
Total (N)	12,053	100
**Sex in the past 12 months**		
Did not have sex in the past 12 months	2694	33.1[31.9-34.3]
Had sex in the past 12 months	5752	66.9[65.7-68.1]
Total (N)	8446	100
**Number of sexual partners in the past 12 months**		
0	2694	33.4[32.2-34.6]
1	5487	64.7[63.5-65.9]
2	167	1.9[1.6-2.2]
Total (N)	8348	100
**Condom use at last sex in the past 12 months**		
Used condom at last sex in past 12 months	363	4.3[3.9-4.9]
Did not use a condom at last sex in past 12 months	5157	61.7[60.4-62.9]
No sex in the past 12 month	2694	34[32.8-35.2]
Total (N)	8,214	100
**First sex before 15 years of age**		
First sex at 15+ years of age	10,978	91.6[90.9-92.1]
First sex before 15 years of age	927	8.4[7.9-9.1]
Total (N)	11,905	100
**Used a condom at last non-marital sexual intercourse**		
Did not use condom	576	78.9[75.3-82.1]
Used condom	169	21.1[17.9-24.7]
Total (N)	745	100
**Ever tested for HIV**		
Never tested	3369	26.4[25.5-27.3]
Ever tested	8654	73.6[72.7-74.5]
Total (N)	12,023	100
**HIV status**		
HIV negative	11,138	95.9 [95.4, 96.3]
HIV positive	461	4.1 [3.7, 4.6]
Total (N)	11,599	100
**Knows HIV status**		
No	78	16.7 [13.1-21.0]
Yes	378	83.3 [79.0-86.9]
Total (N)	456	100
**On ART**		
No	12	3.6 [2.0-6.5]
Yes	366	96.4 [93.5-98.0]
Total (N)	378	100
**Virally suppressed**		
No	127	28.3 [23.8-33.3]
Yes	315	71.7 [66.7-76.2]
Total (N)	442	100
**Overall: knows HIV status, on ART, virally suppressed (among women who are HIV-positive)**		
No	141	30.7 [26.1, 35.6]
Yes	315	69.3 [64.4, 73.9]
Total (N)	456	100
**Female genital cutting status**		
No	4790	41.0[40.0-42.1]
Yes	6576	59.0[57.9-60.0]
Total	11,366	100
**Prevalence of experiencing sexual violence in the first time**		
No	6636	96.0[95.4-96.5]
Yes	237	4.0[3.5-4.6]
Total	6873	100
**Lag: recent/long-term infection**		
Long term	455	99.1[97.8-99.6]
Recent	6	0.9[0.4-2.2]
Total	461	100
**Duration since the first HIV-positive test**		
Less than 1 year	15	3.3[1.8-6.1]
1-5 years	120	33.7[28.4-39.5]
6-10 years	164	45.6[39.9-51.5]
More than 10 years	67	17.3[13.4-22.2]
Total	366	100

⁎ Weighted percent.

### HIV prevalence by demographic and behavioral characteristics

**Figure 2:**
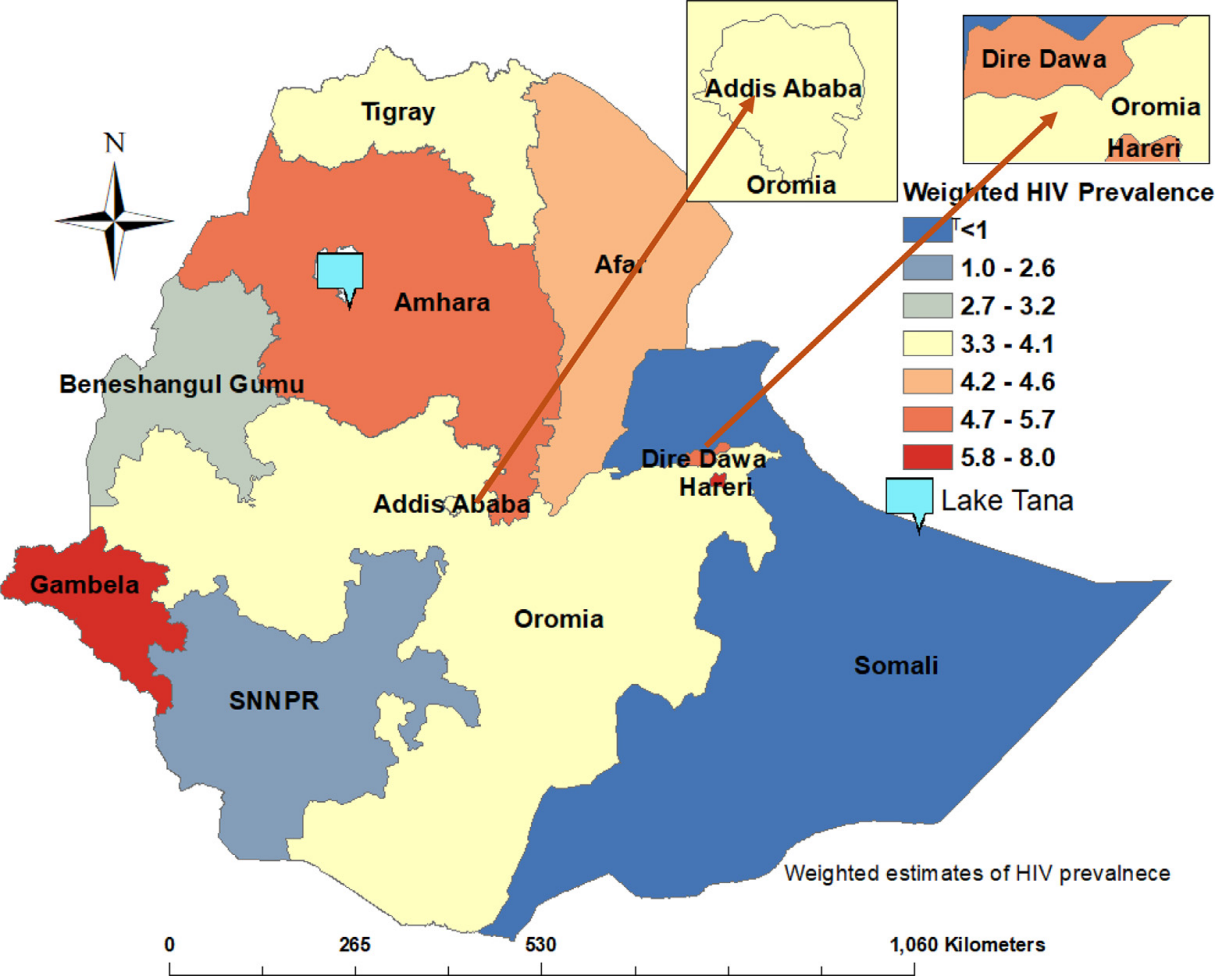
The prevalence of HIV among women in urban Ethiopia by administrative region, Ethiopia Population-based HIV Impact Assessment 2017-2018.

Overall, HIV prevalence among women was 4.1%. By region, HIV prevalence among women was the highest in Gambella (8%), followed by Harari and Dire Dawa, 7.6% and 5.7%, respectively (Figure 2) (Table 2). HIV prevalence was highest among widowed women (15.1%), followed by those who were divorced or separated (8.6%), and lowest among those who had not been married (1.2%) (Table 2). The prevalence was 6.2% among women with no education, 5.1% among those who completed primary school, 3.2% among those who completed secondary school, and 1.1% among those who had education beyond secondary school. Women in the highest wealth quintile had significantly less HIV seropositivity (3.0%) than women in the four lower wealth quintiles, for whom the HIV prevalence rates were 4.6%, 4.5%, 4.6%, and 4.4% for quintiles 1-4, respectively, *P* = 0.029 (Table 2). The prevalence was higher among those employed in the last 12 months (5.1%) than those unemployed in the last 12 months (3.5%). Women reporting first sex before 15 years of age had significantly higher HIV seropositivity (7.6%) than women with first sex at 15+ years of age (3.8%), *P* <0.001 (Table 2). These variables were all significantly associated with HIV prevalence (*P* <0.05) (Table 2).

**Table 2:** HIV status among women aged 15-64 years by selected demographic and behavioral characteristics, Ethiopia Population-based HIV Impact Assessment 2017-2018.

Characteristics	HIV status		N	*P*-value*
	Negative % (95% CI)⁎⁎	Positive % (95% CI)⁎⁎		
**Region**				
Tigray	95.9 [94.3-97.0]	4.1 [3.0-5.7]	914	<0.001
Afar	95.4 [92.9-97.0]	4.6 [3.0-7.1]	481	
Amhara	94.6 [93.3-95.6]	5.4 [4.4-6.7]	1785	
Oromia	95.9 [95.1-96.6]	4.1 [3.4-4.9]	2735	
Somali	99 [97.7-99.5]	1 [0.5-2.3]	570	
Benishangul Gumuz	96.8 [94.6-98.1]	3.2 [1.9-5.4]	455	
SNNPR	97.4 [96.4-98.1]	2.6 [1.9-3.6]	1447	
Gambella	92 [88.8-94.4]	8 [5.6-11.2]	419	
Harari	92.4 [89.2-94.7]	7.6 [5.3-10.8]	432	
Addis Ababa	96.1 [95.0-96.9]	3.9 [3.1-5.0]	1841	
Dire Dawa	94.3 [91.7-96.1]	5.7 [3.9-8.3]	520	
**Age Group of adults**				<0.001
15-24 years	99.2 [98.8-99.4]	0.8 [0.6-1.2]	4788	
25-34 years	95.7 [94.9-96.4]	4.3 [3.6-5.1]	3421	
35-44 years	91.2 [89.7-92.5]	8.8 [7.5-10.3]	1837	
45-54 years	93.2 [91.2-94.8]	6.8 [5.2-8.8]	910	
55-64 years	96.6 [94.7-97.9]	3.4 [2.1-5.3]	643	
**Marital status**				<0.001
Never married	98.8 [98.3-99.1]	1.2 [0.9-1.7]	3788	
Married or living together	96.9 [96.3-97.4]	3.1 [2.6-3.7]	5623	
Divorced or separated	91.4 [89.6-92.9]	8.6 [7.1-10.4]	1394	
Widowed	84.9 [81.7-87.6]	15.1 [12.4-18.3]	717	
**Education level**				<0.001
No education	93.8 [92.4-95.0]	6.2 [5.0-7.6]	1959	
Primary	94.9 [94.1-95.6]	5.1 [4.4-5.9]	4295	
Secondary	96.8 [96.0-97.4]	3.2 [2.6-4.0]	3096	
More than secondary	98.9 [98.3-99.3]	1.1 [0.7-1.7]	2203	
**Wealth quintile**				0.029
Lowest	95.4 [94.1-96.4]	4.6 [3.6-5.9]	1945	
Second	95.5 [94.3-96.5]	4.5 [3.5-5.7]	1964	
Middle	95.4 [94.3-96.3]	4.6 [3.7-5.7]	2255	
Fourth	95.6 [94.6-96.4]	4.4 [3.6-5.4]	2538	
Highest	97.1 [96.3-97.7]	2.9 [2.3-3.7]	2897	
**Employment in the last 12 months**				<0.001
Did not work in the last 12 last months	96.5 [96.0-96.9]	3.5 [3.1-4.0]	7646	
Worked in the last 12 last months	94.9 [94.1-95.6]	5.1 [4.4-5.9]	3934	
**Number of sexual partners in the past 12 months**				<0.001
0	92.1 [90.8-93.2]	7.9 [6.8-9.2]	2572	
1	96.6 [96.0-97.1]	3.4 [2.9-4.0]	5230	
2 or more	88.8 [82.3-93.2]	11.2 [6.8-17.7]	158	
**Condom use at last sex in past 12 months**				<0.001
Used condom at last sex in past 12 months	82.3 [77.0-86.5]	17.7 [13.5-23.0]	343	
Did not use condom at last sex in past 12 months	97.3 [96.8-97.8]	2.7 [2.2-3.2]	4916	
No sex in the past 12 months	92.1 [90.8-93.2]	7.9 [6.8-9.2]	2572	
**First Sex Before 15 years of age**				<0.001
First sex at 15+ years of age	96.2 [95.7-96.6]	3.8 [3.4-4.3]	10477	
First sex before 15 years of age	92.4 [90.2-94.2]	7.6 [5.8-9.8]	886	
**Ever tested for HIV?**				<0.001
Never tested	99 [98.4-99.3]	1 [0.7-1.6]	3221	
Ever tested	94.8 [94.2-95.3]	5.2 [4.7-5.8]	8250	
**Ever had sex**				<0.001
Never had sex	99.4 [99.0-99.7]	0.6 [0.3-1.0]	2806	
Have had sex	94.9 [94.4-95.4]	5.1 [4.6-5.6]	8694	
**Sex in the past 12 months**				<0.001
Did not have sex	92.1 [90.8-93.2]	7.9 [6.8-9.2]	2572	
Had sex	96.4 [95.8-96.9]	3.6 [3.1-4.2]	5479	
**Female genital cutting status**				0.022
No	96.6 [95.9-97.1]	3.4 [2.9-4.1]	4557	
Yes	95.6 [94.9-96.1]	4.4 [3.9-5.1]	6305	
**Experienced sexual violence in the first time**				<0.001
No	95.7 [95.1-96.2]	4.3 [3.8-4.9]	6271	
Yes	88.3 [83.1-92.0]	11.7 [8.0-16.9]	233	

⁎ *P*-values are calculated using the chi-square test.

⁎⁎ Weighted percent.

**Figure 3:**
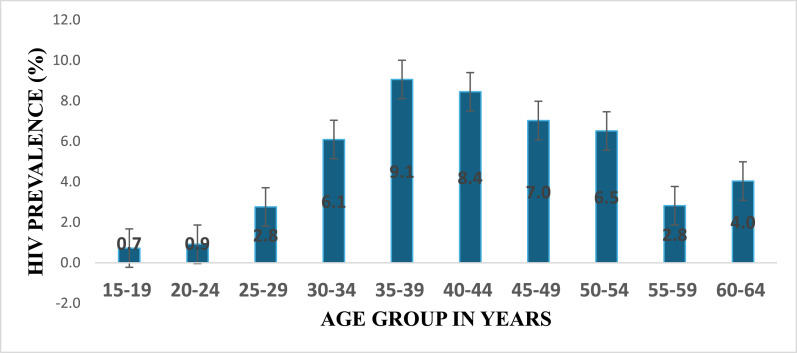
HIV prevalence among women by age categories, Ethiopia population-based HIV Impact Assessment 2017-2018.

The prevalence of HIV among adult women increased with increasing age, reaching a peak (9.1%) in the 35-39 years age group (Figure 3). The prevalence was significantly higher among those who ever had sex (5.1%) than those reporting they had never had sex (0.6%), *P* <0.001 (Table 2). HIV prevalence was significantly lower in women reporting one sexual partner in the past 12 months (3.4%) than women with no sexual partners (7.9%) or women with two or more sexual partners (11.2%) in the past 12 months (*P* <0.001) (Table 2). The prevalence was higher among women who had undergone circumcision (4.4%) than those who had not (3.4%), with a *P*-value of 0.022 (Table 2). The prevalence was significantly higher at 11.7% among those who experienced sexual violence than 4.3% among those who had not, with a *P* <0.001 (Table 2).

### Factors associated with HIV prevalence

The bivariable logistic regression analysis (Table 3) shows that administrative region, age group, marital status, educational status, ever having had sex, age at first sexual encounter, and female genital cutting were significantly associated with HIV seropositivity.

**Table 3:** Bivariate and multivariate logistics regression analysis of independent factors on HIV prevalence among women aged 15-64 years, EPHIA 2017/2018.

Characteristics	COR (95%CI)	*P*-value	AOR (95%CI)	*P*-value
Region				
Somali (Ref.)	1		1	
Tigray	4.1*** (1.8 - 9.7)	0.001	1.9(0.5 - 6.6)	0.332
Afar	4.7*** (1.8 - 12.2)	0.002	2.2(0.5 - 8.7)	0.280
Amhara	5.5*** (2.5 - 12.0)	0.000	2.9*(0.9 - 9.1)	0.075
Oromia	4.1*** (1.8 - 9.1)	0.001	1.8(0.6 - 5.8)	0.315
Benishangul Gumuz	3.2*** (1.4 - 7.2)	0.006	3.1*(1.0 - 10.1)	0.058
SNNPR	2.6** (1.1- 6.0)	0.027	1.1(0.3 - 3.7)	0.863
Gambella	8.3*** (3.2 - 22.1)	0.000	6.2***(1.7 - 23.4)	0.007
Harari	7.9*** (3.6 - 17.3)	0.000	2.6(0.8 - 9.0)	0.128
Addis Ababa	3.9*** (1.8 - 8.6)	0.001	2.1(0.7 - 6.7)	0.215
Dire Dawa	5.9*** (2.3 - 15.0)	0.000	2.2(0.5 - 8.8)	0.273
Age group				
15-24 years (Ref.)	1		1	
25-34 years	5.4*** (3.8 - 7.7)	0.000	3.6***(2.0 - 6.4)	0.000
35-44 years	11.7*** (8.0 - 17.0)	0.000	7.2***(3.9 - 13.5)	0.000
45-54 years	8.8*** (5.5 - 14.1)	0.000	3.6***(1.7 - 7.3)	0.001
55-64 years	4.2*** (2.4 - 7.2)	0.000	0.9(0.3 - 2.5)	0.828
Sex of head of household				
Male headed (Ref.)	1		1	
Female headed	1.9*** (1.5 - 2.4)	0.000	1.3(0.9 - 1.9)	0.163
Marital status				
Never married (Ref.)	1		1	
Married or living together	2.6*** (1.8 - 3.7)	0.000	0.7(0.4 - 1.3)	0.246
Divorced or separated	7.5*** (5.0 - 11.2)	0.000	1.5(0.8 - 2.9)	0.227
Widowed	14.1*** (9.2 - 21.8)	0.000	3.4***(1.6 - 7.0)	0.001
Education level				
No education	5.7*** (3.6 - 9.2)	0.000	4.7***(2.2 - 10.2)	0.000
Primary education	4.7*** (2.9 - 7.5)	0.000	5.6***(2.8 - 11.2)	0.000
Secondary	2.9*** (1.8 - 4.6)	0.000	4.4***(2.2 - 9.2)	0.000
More than secondary (Ref.)	1		1	
Ever had sex				
No (Ref.)	1		1	
Yes	9.6*** (5.3 - 17.4)	0.000	2.6*(0.9 - 7.3)	0.077
Female genital cutting status				
Had no genital cutting (Ref.)	1		1	
Had genital cutting	1.3** (1.1 - 1.6)	0.014	3.4***(2.0 - 5.8)	0.000
Experienced sexual violence in the first time				
No (Ref.)	1			
Yes	3.0*** (1.9 - 4.6)	0.000	0.8*(0.6 - 1.0)	0.096

Adjusted odds ratio = AOR; Ref. = reference category, crude odds ratio = COR.

* *P* <0.05; ** *P* <0.001.

In the multivariable regression analysis, HIV seropositivity among women was significantly associated with administrative region, age, marital status, educational level, and female genital cutting. Women from the Somali region had significantly lower odds of HIV seropositivity than women from all other regions (Table 3). Multivariable logistic regression showed that the adjusted odds of HIV seropositivity rates were 3.6, 7.2, and 3.6 times higher for women aged 25-34 years, 35-44 years, and 45-54 years, respectively, than women aged 15-24 years, *P* <0.001 (Table 3). Women with higher than secondary education had significantly lower HIV prevalence than women with lower education. The adjusted odds ratios (AORs) of HIV seropositivity were 4.7 (95% CI 2.2-10.2), 5.6, (95% CI 2.8-11.2), and 4.4 (95% CI 2.2-9.2) times higher for women with no education, primary education, and secondary education, respectively, than women with higher than secondary education, *P* <0.001 (Table 3). The odds of HIV seropositive among widows was 3.4 (95% CI 1.6-7.0) times higher than among women who had never married. The odds of HIV seropositivity among women who underwent genital cutting was 3.4 times higher (95% CI 2.0-5.8) than for those who did not undergo genital cutting, with a *P* <0.001 (Table 3).

## Discussion

HIV prevalence among urban Ethiopian women was 4.1%, with higher rates in Gambella, Harari, and Dire Dawa, and among widowed women, those with low education, early sexual debut, or multiple partners. Risk increased with age, peaking at 35-39 years, and was strongly linked to marital status, education, and female genital cutting. These patterns reflect broader social determinants structural factors like education and wealth, gendered power imbalances, and social vulnerability, which shape exposure and access to care. Together, they underscore the need for structural interventions alongside biomedical strategies, with targeted efforts for vulnerable subgroups and high-prevalence regions.

The HIV prevalence among women aged 15-64 years in urban Ethiopia of 4.1% is comparable with the finding from other African countries such as Côte d’Ivoire (4.4%) [20], Cameroon (5.5%) [21], and Rwanda (6.5%) [22] and lower than studies from Uganda (9.8%) [23], Namibia (13.2%) [24], Zimbabwe (16.4%) [25], Malawi (17.7%) [26], and Zambia (18.7%) [27]. Several studies conducted among non-pregnant women in parts of east and southern Africa describe prevalence rates ranging from 14.5% in East Africa to 38.7% in Lusaka, (Zambia) and 39.5% in Durban, South Africa [28]. This variation among the different studies in similar settings may be explained by the variation in the overall burden of HIV among the general population across these settings as well as possible differences in demographic and behavioral factors [[29], [30], [31]].

The findings from our analysis indicate that females with the highest HIV prevalence were aged 35-44 years compared with women aged 15-24 years. This observation is similar to what has been reported by other studies [32,33], in which women who belonged to the older age group were significantly more likely to have HIV infection than the younger age groups in Lesotho in 2019 [34], Tanzania in 2015 [32], and South Africa in 2021 [31]. The higher burden of HIV among older women could reflect cumulative past infections, survival on ART, generational prevention gaps, and partner dynamics, highlighting the need for targeted, age-specific interventions.

Our results also show that women with higher education levels were less likely to have high HIV prevalence than those who were less educated or had no education. Those with no formal education had 4.7 times the odds of HIV seropositivity (AOR 4.7; 95% CI 2.2-10.2) compared with those who had higher than secondary school education, whereas women who had secondary school education had 4.4 times the odds of being HIV seropositive (AOR 4.4; 95% CI 2.2-9.2) compared with those who achieved higher than secondary school education. Our finding that HIV in Ethiopian women is inversely associated with education level is consistent with the results of studies in other countries [30,35,36].

Ethiopian schools provide instruction about HIV; having such information could be lifesaving for women and, subsequently, for their children. Education may help to delay sexual debut and early marriage among girls. In addition, women with higher education may be able to earn an income; be less financially dependent on their husbands; and, ultimately, more capable of insisting on safe sex practices, monogamous marriage, and marital arrangements involving HIV testing.

A small proportion of women positive for HIV did not report ever having had sex. In this group of women, HIV infection may have occurred through nonsexual means, such as vertical transmission or traditional body modification procedures, such as female genital mutilation, uvulectomy, tattooing, scarification, or piercing. Their responses also could be due to social desirability bias and based on survey participants’ belief that their answers reflect on them as a person. For the older women with no education or limited education without access to school-based HIV education, HIV education programs should be expanded to include less educated and possibly illiterate women.

### Limitations

This study has several limitations. First, the analysis includes only urban women younger than 65 years interviewed in EPHIA, excluding rural women and those aged 65 years and older. Second, measuring “ever having sex” as a risk factor for HIV acquisition is challenging. Third, accurate reporting of sexual and gender-based violence is difficult given its sensitive nature. Fourth, although interviewers were trained to ensure confidentiality, surveys were conducted in the participants’ homes. Finally, some women may have been reluctant to disclose sensitive sexual behaviors due to embarrassment or fear of being overheard by family members.

## Conclusion

In this secondary analysis of the EPHIA survey, HIV prevalence among urban women aged 15-64 years was 4.1%, with significantly higher odds of infection among women aged 35-44 years, widows, and women with limited education. Our findings indicate that HIV prevention, care, and treatment programs in Ethiopia should prioritize specific groups, including women aged 35 years and older, non-pregnant women, and those with limited access to services, particularly, those with low education. Targeted efforts to encourage widowed women to undergo HIV testing would be valuable. Prevention programs aimed at reducing new infections among urban women should be enhanced, with a focus on awareness-raising and behavioral transformation measures. In addition, strategies to increase awareness among less educated women or those with no education are essential to ensure comprehensive support for all vulnerable populations.

These findings hold significant potential to guide evidence-based programmatic decisions within the country. They highlight opportunities to strengthen existing systems for targeted HIV testing, including testing for current and former spouses of individuals positive for HIV and widowed and non-pregnant women. Implementing these actions could substantially improve the effectiveness of HIV prevention and care initiatives.

## Availability of data and materials

Data are for public release and can be accessed through the Population-based HIV Impact Assessment Data Manager website at https://phia-data.icap.columbia.edu/datasets?country_id=12.

## Declaration of competing interest

The authors have no competing interests to declare.

## References

[1] UNAIDS. Global HIV statistics, FACT SHEET, 2025.

[2] UNAIDS. HIV and adolescent girls and young women, 2024.

[3] UNAIDS. Global AIDS Update. https://www.unaids.org/en/resources/documents/2024/global-aids-update-2024; 2024. [accessed 5 May 2026].

[4] World Health Organization. Global Health Observatory (GHO) data - HIV: estimated number of people living with HIV. https://www.who.int/data/gho/data/indicators/indicator-details/GHO/estimated-number-of-people–living-with-hiv; 2024. [accessed 5 May 2026].

[5] Cassim N., Coetzee L.M., da Silva M.P., Glencross D.K., Stevens WS. (2023). Assessing very advanced HIV disease in adolescent girls and young women. South Afr J HIV Med.

[6] Central Statistical Agency (CSA), The DHS Program I.M.B. COMMUNITY FOUNDATION. Ethiopia Demographic and Health Survey (EDHS), 2025.

[7] Tsega E., Mengesha B., Nordenfelt E., Hansson B.G., Lindberg J. (1988). Serological survey of human immunodeficiency virus infection in Ethiopia. Ethiop Med J.

[8] Mehret M., Khodakevich L., Zewdie D., Ayehunie S., Gizaw G., Shanko B. (1990). HIV-1 infection and related risk factors among female sex workers in urban areas of Ethiopia. Ethiop J Health Dev.

[9] (HAPCO). EHAPCO, (GAMET) GHAMaET. HIV /AIDS IN ETHIOPIA AN EPIDEMIOLOGICAL SYNTHESIS World Bank Global HIV/AIDS Program, Program WBGHA; 2008.

[10] Mabala R. (2006). From HIV prevention to HIV protection: addressing the vulnerability of girls and young women in urban areas. Environ Urb.

[11] Kalichman SC. (1998). Influencing HIV transmission risk. Focus.

[12] Sia D., Onadja Y., Hajizadeh M., Heymann S.J., Brewer T.F., Nandi A. (2016). What explains gender inequalities in HIV/AIDS prevalence in sub-Saharan Africa? Evidence from the demographic and health surveys. BMC Public Health.

[13] UNAIDS S. Transactional sex and HIV risk: from analysis to action, 2018.

[14] Sachathep K., Radin E., Hladik W., Hakim A., Saito S., Burnett J. (2021). Population-based HIV Impact assessments survey methods, response, and quality in Zimbabwe, Malawi, and Zambia. J Acquir Immune Defic Syndr 1999.

[15] (EPHI) EPHI. Ethiopia Population-based HIV Impact Assessment (EPHIA) 2017-2018. 2020.

[16] Patel H.K., Duong Y.T., Birhanu S., Dobbs T., Lupoli K., Moore C. (2021). A comprehensive approach to assuring quality of laboratory testing in HIV surveys: lessons learned from the population-based HIV Impact assessment project. J Acquir Immune Defic Syndr.

[17] Low A., Thin K., Davia S., Mantell J., Koto M., McCracken S. (2019). Correlates of HIV infection in adolescent girls and young women in Lesotho: results from a population-based survey. Lancet HIV.

[18] Kimanga D.O., Ogola S., Umuro M., Ng'ang'a A., Kimondo L., Murithi P. (1999). Prevalence and incidence of HIV infection, trends, and risk factors among persons aged 15-64 years in Kenya: results from a nationally representative study. J Acquir Immune Defic Syndr.

[19] StataCorp (2015).

[20] (MSHP) MdlSedlHP (2021).

[21] Ministry of Health (MOH) (2020).

[22] (RBC) RBC. Rwanda population-based HIV Impact assessment (RPHIA) 2018-2019. 2020.

[23] (MOH) (2019).

[24] Okango E., Mwambi H., Ngesa O. (2016). Spatial modeling of HIV and HSV-2 among women in Kenya with spatially varying coefficients. BMC Public Health.

[25] Ministry of Health, Child C (MoHCC) (2019).

[26] MPHIA. Malawi population-based HIV. Impact Assess 2018:2015-6.

[27] Ministry of Health. Z (2016).

[28] Ramjee G., Daniels B. (2013). Women and HIV in sub-Saharan Africa. AIDS Res Ther.

[29] Ekholuenetale M., Onuoha H., Ekholuenetale C.E., Barrow A., Nzoputam CI. (2021). Socioeconomic inequalities in human immunodeficiency virus (HIV) sero-prevalence among women in Namibia: further analysis of population-based data. Int J Environ Res Public Health.

[30] Hamda S.G., Tshikuka J.G., Joel D., Monamodi G., Masupe T., Setlhare V. (2020). Sociodemographic predictors of HIV infection among pregnant women in Botswana: cross-sectional study at 7 health facilities. J Int Assoc Providers AIDS Care.

[31] Mabunda S.A., Sigovana K., Chitha W., Apalata T., Nomatshila S. (2021). Socio-demographic associations of HIV among women attending antenatal care in selected rural primary care facilities in South Africa's Eastern Cape Province. BMC Infect Dis.

[32] Singh R.K., Patra S. (2015). What factors are responsible for higher prevalence of HIV infection among urban women than rural women in Tanzania?. Ethiop J Health Sci.

[33] Zuma K., Gouws E., Williams B., Lurie M. (2003). Risk factors for HIV infection among women in Carletonville, South Africa: migration, demography and sexually transmitted diseases. Int J STD AIDS.

[34] (MOH). MoH. Lesotho population-based HIV impact assessment 2016-7. 2019.

[35] David J.K., Pant R., Allam R.R., Priya V.M.P., Aridoss S., Arumugam E. (2020). The relationship between educational attainment and HIV prevalence among pregnant women attending antenatal clinics in six states of India: sentinel surveillance from 2010 to 2017. Indian J Public Health.

[36] Bago J.L., Ouédraogo E., Lompo ML. (2021). HIV among women: does education matter more than we previously thought?. Stud Econ Econ.

